# Immortalized murine tenocyte cells: a novel and innovative tool for tendon research

**DOI:** 10.1038/s41598-023-28318-4

**Published:** 2023-01-28

**Authors:** Gil Lola Oreff, Barbara Maurer, Ahmed N. ELKhamary, Iris Gerner, Veronika Sexl, Florien Jenner

**Affiliations:** 1 Veterinary Tissue Engineering and Regenerative Medicine Lab, Equine Surgery Unit, Department of Companion Animals and Horses, University of Veterinary Medicine Vienna, Veterinaerplatz 1, 1210 Vienna, Austria; 2 Institute of Pharmacology and Toxicology, Department of Biomedical Sciences, University of Veterinary Medicine Vienna, Veterinaerplatz 1, 1210 Vienna, Austria

**Keywords:** Senescence, Tendons

## Abstract

Primary tenocytes rapidly undergo senescence and a phenotypic drift upon in vitro monolayer culture, which limits tendon research. The *Ink4a/Arf* locus encodes the proteins p16^Ink4a/Arf^ and p14^ARF^ (p19^ARF^ in mice) that regulate cell cycle progression and senescence. We here established an immortalized cell line using tenocytes isolated from *Ink4a/Arf deficient* mice (*Ink4a/Arf*^*−*/*−*^). These cells were investigated at three distinct time points, at low (2–5), intermediate (14–17) and high (35–44) passages. Wild-type cells at low passage (2–5) served as controls. *Ink4a/Arf*^*−*/*−*^ tenocytes at all stages were comparable to wild-type cells regarding morphology, expression of tenogeneic genes (*collagen type 1, 3 and 5, Scleraxis, Tenomodulin and Tenascin-C*), and surface markers (CD29, CD44 and CD105) and form 3D tendon-like structures. Importantly, *Ink4a/Arf*^*−*/*−*^ tenocytes maintained their phenotypic features and proliferation potential in culture for more than 40 passages and also following freeze–thaw cycles. In contrast, wild-type tenocytes underwent senescence starting in passage 6. These data define *Ink4a/Arf*^*−*/*−*^ tenocytes as novel tool for in vitro tendon research and as valuable in vitro alternative to animal experiments.

## Introduction

Tendinopathy is the most common musculoskeletal complaint for which patients seek medical attention^1–5^. It is prevalent in occupational and athletic settings, afflicting 25% of the adult population and accounting for 30–50% of sport injuries^2^. The incidence is rising in line with major risk factors, including repetitive overuse due to mechanically demanding work and sports activities, age, obesity and diabetes^5^. Thus, tendon injuries pose a substantial socioeconomic burden and an escalating challenge to healthcare services, with the annual health expenditure exceeding €145 billion^1–5^.

Due to its low cellularity, poor vascularity and bradytrophic metabolism, a tendon’s response to injury is inefficient and results in a fibrovascular scar that never attains the gross, histological or mechanical characteristics of a normal tendon, leading to high re-injury rates and chronic tendinopathy^2,4^. Current treatment fails to restore the functional properties of injured tendons, significantly decreasing mobility, productivity and quality of life^5–7^. In an increasingly active and ageing population, the long-term morbidity of tendon disease represents a growing unmet clinical need. However, research efforts are hampered by the low yield of primary tenocytes, their limited proliferation rate and lifespan in vitro and the corresponding lack of relevant in vitro models^8,11^. Tenocytes in culture undergo senescence and a phenotype drift, losing their fusiform morphology and the expression of typical lineage markers such as *Collagen1 (Col1)*, *Tenomodulin (Tnmd)*, *Scleraxis (Scx)* and *Tenascin C (Tnc)*^10,12–14^ at passage 3–5^10–12,14^, which restricts the number and quality of cells available for research and thereby experimental design. The resulting need to sacrifice high numbers of animals to obtain sufficient tenocytes for research poses ethical problems. Donor variability further increases the number of animals needed to differentiate inter-individual variability from the effects caused by experimental conditions^15^. Efforts to immortalize tenocytes by delivering the oncogene SV40 large T antigen to murine tenocytes using a piggyBac transposon system successfully prolonged the lifespan of the cells to passage 30 but drastically impaired tendon marker expression (*Col1, Col3, Scx, Tnmd* and *Tnc*)^10^. In contrast, three-dimensional culture in collagen gels improves proliferation rates, tendon marker expression and cellular yield compared to 2D culture but does not overcome the problem of senescence^10^. Thus, to date, in vitro tendon research possibilities are limited, and better research tools are urgently required to advance the development of satisfactory treatment options.

The cell cycle regulators p16^Ink4a/Arf^ and p14^ARF^ (p19^ARF^ in mice) are encoded by the *Ink4a/Arf* locus. p16^Ink4a/Arf^ is a cyclin-dependent kinase inhibitor that prevents G1–S phase progression. p14^ARF^ influences responses to a wide range of cellular stress signals by interfering with p53–mediated transcription. Both proteins play an essential role in cell growth, survival and senescence and act as tumour suppressors^16–20^. Targeted disruption of the *Ink4a/Arf* locus in mice generates a cancer-prone phenotype^16–21^. Mutations in the *Ink4a/Arf* locus in humans are frequent and found in multiple malignancies, including hematopoietic tumours, melanoma, carcinoma and sarcoma^16^. The expression of p16^Ink4a/Arf^ and p19ARF increases markedly with ageing^22,23^ and contributes to the decline of the replicative potential of stem cells^22^. In the ageing Achilles tendon, upregulation of *Ink4a/Arf* locus leads to exhaustion of the tendon stem cells pool in size and function^24–26^ and may contribute to the decreased healing potential following injury in aged tendons. Deletion of the *Ink4a/Arf* locus has been shown to immortalize various cell types, including MEFs (mouse embryonic fibroblasts)^21,27^.

This study establishes an immortalized tenocyte cell line from Achilles tenocytes isolated from *Ink4a/Arf* deficient mice (*Ink4a/Arf*^*−*/*−*^). The immortalized tenocytes behave like their wild-type primary counterparts in monolayer and 3D culture but in contrast to wild-type primary cells preserve their proliferation potential and tenogenic properties even during long-term culture and after freeze–thaw cycles, thus providing a valuable in vitro alternative to animal models for tendon research.

## Results

### Ink4a/Arf^*−*/*−*^ tenocytes proliferate consistently while maintaining a tenogenic morphology

**Figure 1:**
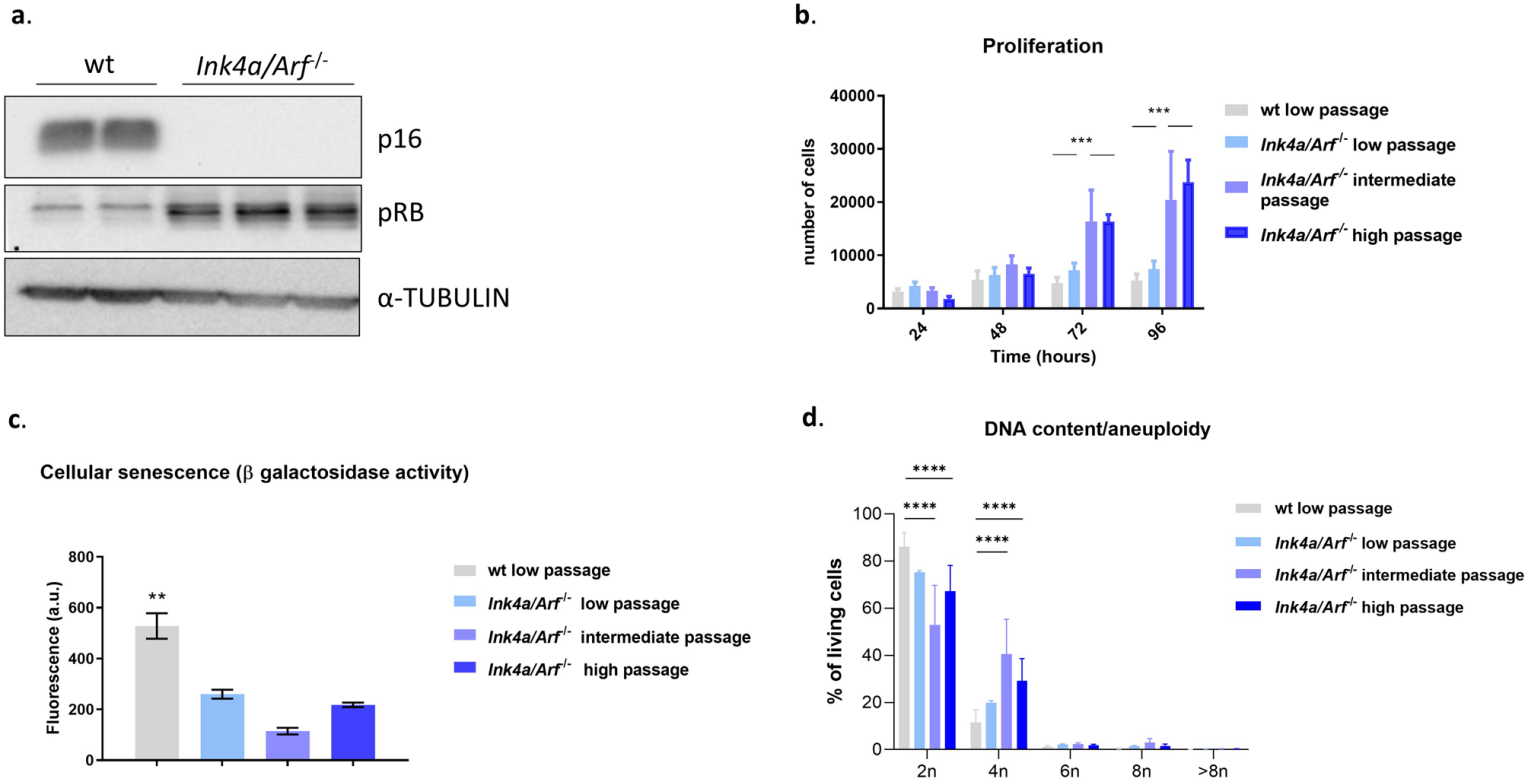
Characterization of *Ink4a/Arf*^*−*/*−*^ compared to wild-type (wt) tenocytes (n = 3 different biological replicates, ** *p*-value < 0.01, *** *p*-value < 0.001, for mean, s.d. and *p*-values see Table 1) (**a**) Western blot of p16 and α-Tubulin (as loading control) in *Ink4a/Arf*^*−*/*−*^ and wt cells confirmed the successful deletion of p16Ink4a/Arf in the *Ink4a/Arf*^*−*/*−*^ cells. (**b**) The proliferation capacity, illustrated as the number of cells obtained over four days, of intermediate and high passages *Ink4a/Arf*^*−*/*−*^ tenocytes was significantly higher than of wt tenocytes. (**c**) Senescence, illustrated as the level of β-galactosidase activity, of *Ink4a/Arf*^*−*/*−*^ tenocytes was significantly lower than in wt tenocytes (a.u.—arbitrary units). (**d**) DNA content/Aneuploidy, intracellular DAPI staining of wt low passage (3 independent donors), *Ink4a/Arf*^*−*/*−*^ low, intermediate and high passage (3 independent cell lines).

Western blot analysis demonstrated p16INK4a in wt tenocytes but a complete loss of p16INK4a protein expression in the *Ink4a/Arf*^*−*/*−*^ cells, confirming the successful deletion of the locus in the *Ink4a/Arf*^*−*/*−*^ tenocytes (Fig. 1a, Supplementary Fig. S1a).

**Figure 2:**
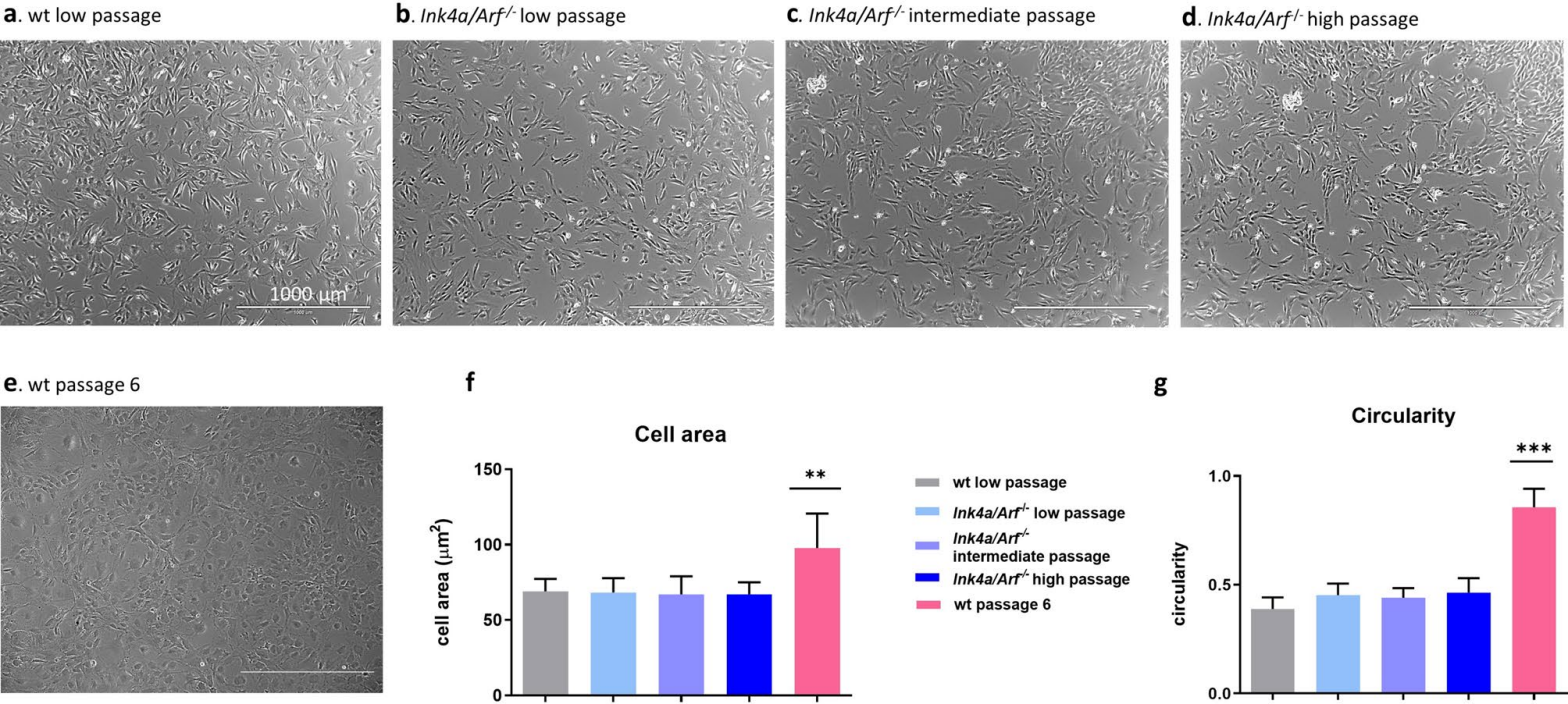
Morphology, cell area and circularity of *Ink4a/Arf*^*−*/*−*^ cells in comparison to wild-type (wt) cells in different passages. **p-value < 0.01, ***p-value < 0.001. **a**–**e** wt tenocytes in a low passage (**a**, passage 2–5) and *Ink4a/Arf*^*−*/*−*^ tenocytes in a low (**b**, passage 2–5), intermediate (**c**, passage 14–17) and high (**d**, passage 35–44) passage showed similar tenogenic morphology while in wt tenocytes in passage 6 (**e**) phenotypic drift was already apparent. Micrographs were obtained using phase contrast with a 40 × objective (scale bar = 1000 µm). (**f**) Cell area (μm^2^) of the wt cells and the *Ink4a/Arf*^*−*/*−*^ cells in different passages. Wt cells in passage 6 showed significantly higher cell area indicative for dedifferentiation. (**g**): Circularity (0–1 range) of the wt cells and the *Ink4a/Arf*^*−*/*−*^ cells in different passages. Circularity in wt cells in passage 6 was significantly closer to 1 than the rest of the groups suggesting a rounder appearance indicative for dedifferentiation.

*Ink4a/Arf*^*−*/*−*^ tenocytes maintained their proliferation potential up to passage 44, the highest passage included in this study. The proliferation rates of *Ink4a/Arf*^*−*/*−*^ cells at the intermediate and higher passage remained consistently high. *Ink4a/Arf*^*−*/*−*^ tenocytes proliferated even faster than wt controls and *Ink4a/Arf*^*−*/*−*^ cells at a low passage (Fig. 1b, Table 1).

**Table 1:** Summary of proliferation, senescence (β-galactosidase activity), flow cytometry and qPCR (gene expression) analyses performed in wild-type (wt) and *Ink4a/Arf*^*−*/*−*^ tenocytes.

		wt		*Ink4a/Arf*^*−*/*−*^ low passage			*Ink4a/Arf*^*−*/*−*^ intermediate passage			*Ink4a/Arf*^*−*/*−*^ high passage		
		Mean	s.d	Mean	s.d	p value	Mean	s.d	p value	Mean	s.d	p value
Proliferation (number of cells)	24 h	3159	561.1	4056	654.9	0.96	3201	651.8	0.99	1807	626.6	0.88
	48 h	5032	841.9	6338	1407	0.89	7872	1741	0.41	6457	868	0.86
	72 h	4811	1109	7186	1448	0.56	16,342	6600	< 0.0001	16,772	1259	< 0.0001
	96 h	5259	1107	7196	1152	0.71	22,917	9525	< 0.0001	25,785	3153	< 0.0001
Cellular senescence (β galactosidase activity) (Fluorescence a.u)		528.2	122.8	260	42.72	0.008	114.8	31.87	0.0005	218	22.19	0.003
Flow cytometry (% of living cells)	CD29	99.9	0.01	99.93	0.06	0.99	99.56	0.5467	0.29	99.98	0.02082	0.99
	CD105	38.8	35.09	37.6	8.99	0.99	10.05	17.07	0.36	0.2967	0.4712	0.16
	CD90	93.7	1.29	95.28	1,776	0.99	91.95	8,828	0.99	25.79	35.89	0.009
	CD44	98.86	1.54	99.24	0.31	0.95	98.61	0.4388	0.98	99.5	0.5746	0.79
Gene expression 2D (normalized mRNA level)	Collagen 1	8.16	4.6	5.5	5.2	0.9	5.7	5.6	0.9	4.5	4.7	0.8
	Collagen 3	2.2	1.4	0.4	0.3	0.47	1.9	1.5	0.99	1.8	1.7	0.99
	Collagen 5	0.2	0.1	0.04	0.02	0.36	0.09	0.11	0.72	0.09	0.12	0.7
	Tenomodulin	0.08	0.008	0.02	0.03	0.22	0.03	0.04	0.3	0.03	0.04	0.27
	Tenascin C	0.13	0.06	0.03	0.01	0.25	0.07	0.05	0.6	0.07	0.07	0.6
	Scleraxis	0.01	0.01	0.01	0.004	0.9	0.01	0.003	0.8	0.02	0.01	0.99

Results are presented as mean ± s.d. *Ink4a/Arf*^*−*/*−*^ cells (in low, intermediate, and high passages) were compared to wt cells using an ANOVA with Tukey’s multiple comparisons test, and the *p*-value is shown for each evaluation. (a.u.—arbitrary units).

Senescence, measured by ß-Galactosidase activity, remained significantly lower in *Ink4a/Arf*^*−*/*−*^ tenocytes than in wt tenocytes even in high passages (*p*-value < 0.01, Fig. 1c, Table 1). Aneuploidy, examined by flow cytometry of intracellular DAPI staining, was not apparent in *Ink4a/Arf*^*−*/*−*^ tenocytes and DNA content remained constant in high passages (Fig. 1d, Supplementary Fig. S1b). The higher proliferation of *Ink4a/Arf*^*−*/*−*^ tenocytes was visible by a higher fraction of cells in the S phase of the cell cycle (chromosome duplication, 4n; Fig. 1d, Supplementary Fig S1b). Furthermore, low, intermediate, and high passage *Ink4a/Arf*^*−*/*−*^ cells maintained their fusiform morphology and adherence to the plastic plate surface, comparable to wt cells (Fig. 2a–d). In contrast, wt cells changed their morphology at passage six and drifted towards a rounder and larger appearance with less defined margins and a flattened shape indicative for dedifferentiation (Fig. 2e–g).

**Figure 3:**
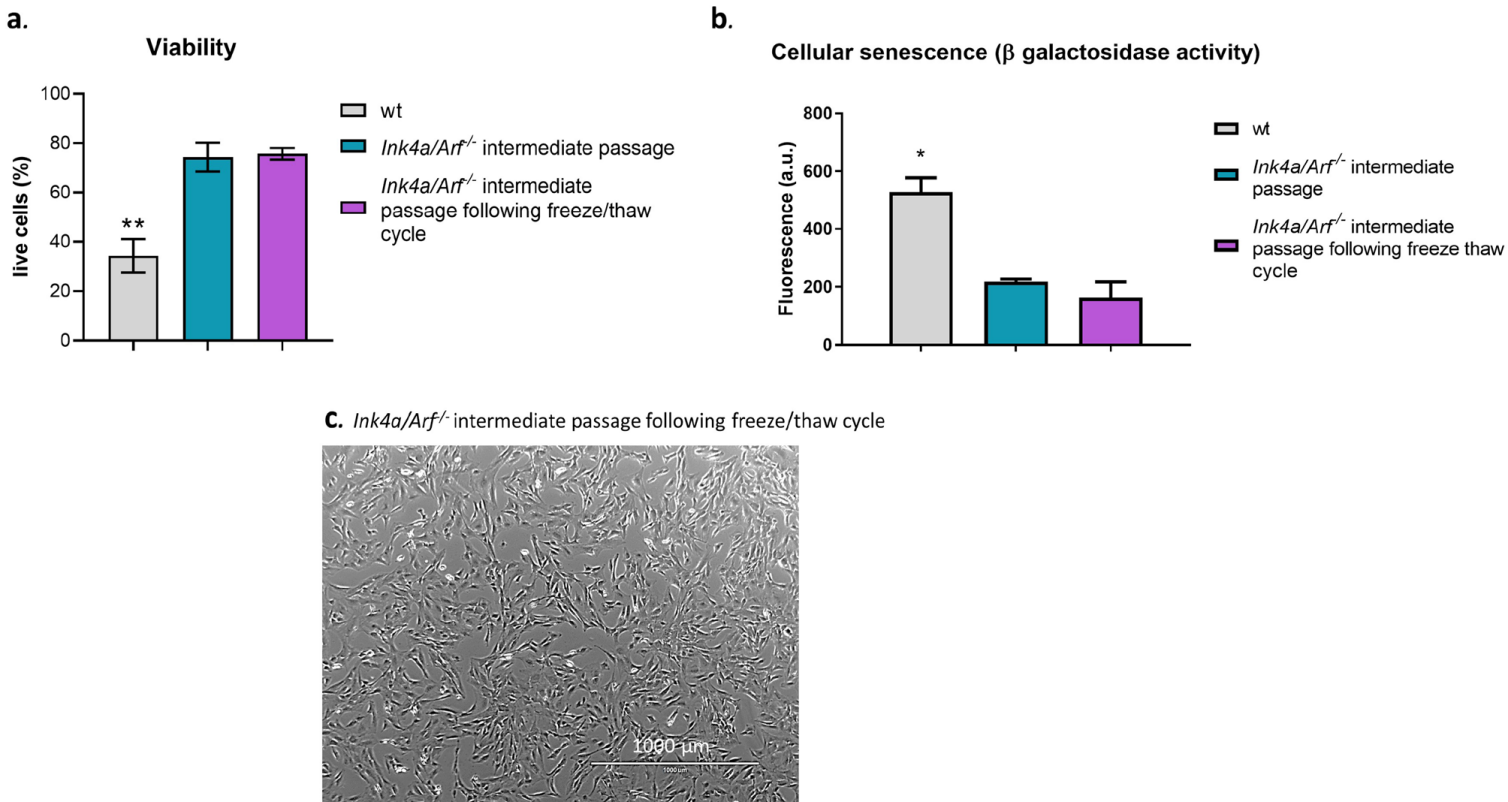
Tenocytes from *Ink4a/Arf*^*−*/*−*^ mice maintain their viability, low senescence and tenogenic morphology following freeze–thaw cycles. *p-value < 0.05, **p-value < 0.01. (**a**) Flow cytometry analysis of Annexin-V staining for low passage wild-type (wt) cells, *Ink4a/Arf*^*−*/*−*^ cells at an intermediate passage and *Ink4a/Arf*^*−*/*−*^ cells at an intermediate passage following a freeze and thaw cycle (n = 3 biological replicates). Wt cells showed significantly lower viability than *Ink4a/Arf*^*−*/*−*^ cells at an intermediate passage with and without a freeze–thaw cycle. (**b**) Senescence, illustrated as the level of β-galactosidase activity, was significantly higher in wt than in *Ink4a/Arf*^*−*/*−*^ cells at intermediate passage and *Ink4a/Arf*^*−*/*−*^ cells at intermediate passage following freeze and thaw cycle (n = 3 different biological replicates) (a.u.—arbitrary units). (**c**) *Ink4a/Arf*^*−*/*−*^ cells at intermediate passage following freeze and thaw cycle showed tenogenic morphology when imaged using phase contrast with a 40 × objective (scale bar = 1000 µm).

### Ink4a/Arf^*−*/*−*^ tenocytes maintain cell viability and tenogenic features following freeze–thaw cycles

Viability of *Ink4a/Arf*^*−*/*−*^ tenocytes was not affected by the freeze–thaw cycle (75.6% ± 4 live cells following a freeze–thaw cycle versus 74.2% ± 9 before freezing, *p*-value = 0.98) and remained significantly higher than the viability of wt cells (34.2% ± 10, *p*-value < 0.01) (Fig. 3a). The freeze–thaw cycle did not induce senescence in *Ink4a/Arf*^*−*/*−*^ cells (115 a.u. ± 32 before freezing, 162 arbitrary units (a.u.) ± 134 after a freeze–thaw cycle, *p*-value = 0.87), as shown by the consistently lower β-galactosidase activity of Ink4a/Arf^*−*/*−*^ compared to wt cells (528 a.u. ± 123, *p*-value < 0.05) (Fig. 3b). Cell morphology remained unchanged, tenocytes maintained their fusiform appearance without any evidence of a rounded or flattened phenotype indicating dedifferentiation (Fig. 3c).

**Figure 4:**
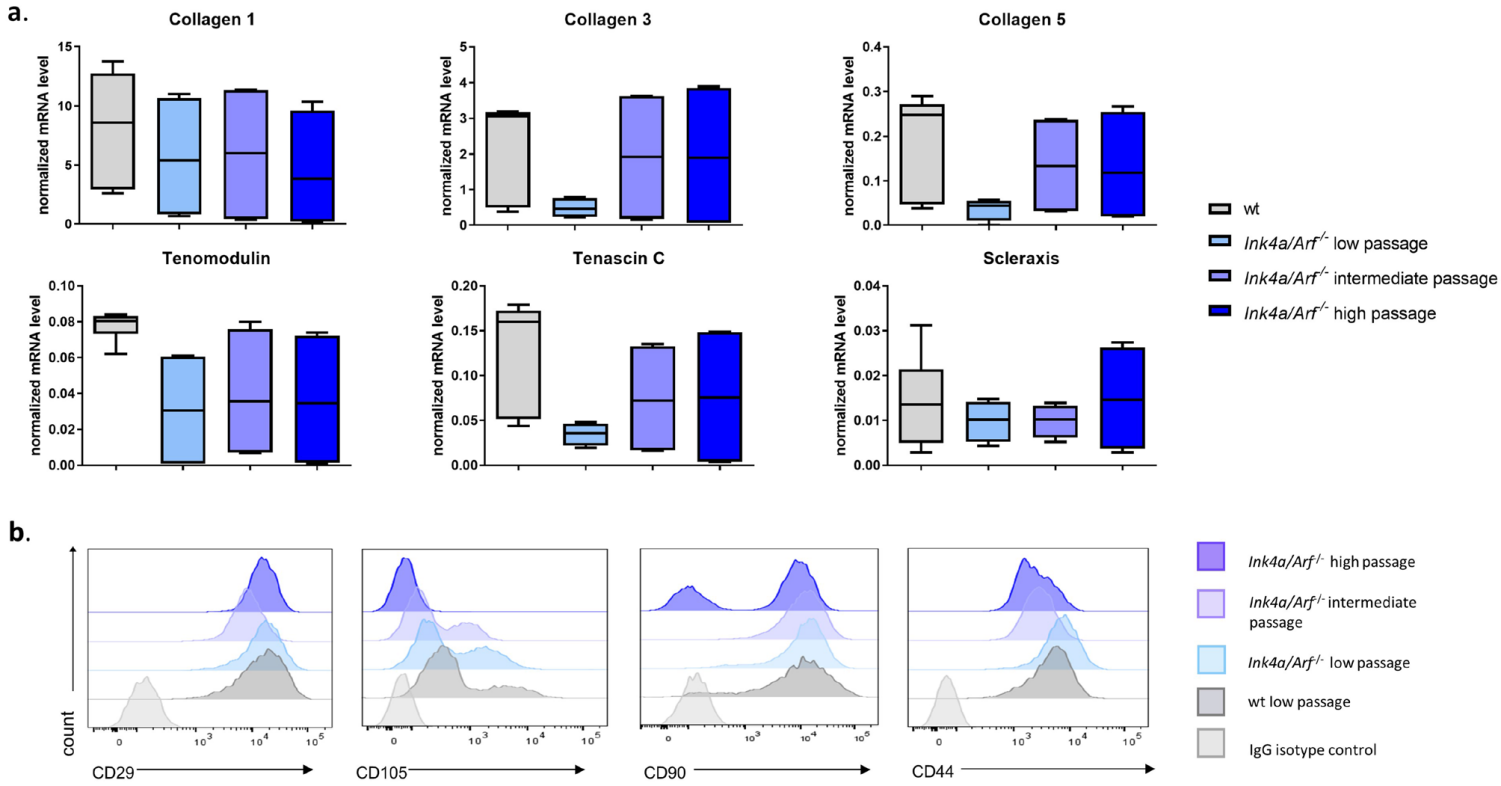
Tenocytes from *Ink4a/Arf*^*−*/*−*^ mice in low, intermediate, and high passage exhibit tenogenic characteristics comparable to tenocytes from wild-type (wt) mice in low passage. (**a**) Box & whiskers plots (mean indicated by the horizontal line and whiskers indicating min–max range) illustrating the qPCR data of tendon markers *Col1, Col3, Col5, Scx, Tnmd* and *Tnc* in wt cells and *Ink4a/Arf*^*−*/*−*^ cells from different passages (n = 3 different biological replicates). For mean normalized mRNA levels, s.d. and *p*-values see Table 1. (**b**) Representative histograms of flow cytometry analysis of CD29, CD105, CD90 and CD44 expression in wt (low passage) and *Ink4a/Arf*^*−*/*−*^ cells (in low, intermediate, and high passages) revealed similar levels of surface markers confirming their tenogenic phenotype (n = 3 different biological replicates).

### Ink4a/Arf^*−*/*−*^ tenocytes maintain tenogenic characteristics in culture

The expression of tenocyte markers (collagen type 1 (*Col1), Col3, Col5, Scleraxis* (*Scx*)*, Tenomodulin* (*Tnmd*) and Tenascin-C (*Tnc*)) expression of *Ink4a/Arf*^*−*/*−*^ cells at low, intermediate, and high passage remained stable at all time points investigated and equivalent to wt cells (Fig. 4a, Table 1). Also the levels of the surface markers CD29, CD90, CD105 and CD44^28^ measured by flow cytometry were comparable between *Ink4a/Arf*^*−*/*−*^ and wt cells (Fig. 4b, Table 1). CD29 and CD44 expression was high (> 95%) in both cell populations and remained high in all *Ink4a/Arf*^*−*/*−*^ passages investigated. CD90 expression was high (> 90%) in wt tenocytes and *Ink4a/Arf*^*−*/*−*^ cells in early and intermediate passages but decreasing in higher passages (*p* = 0.009, Supplementary Fig S2, Table 1). CD105 was expressed in only a small number (38%) of low passage cells of both wt and *Ink4a/Arf*^*−*/*−*^ genotype and in fewer cells in the intermediate (*p*-value = 0.36) and high (p-value = 0.16) passages of *Ink4a/Arf*^*−*/*−*^ cells (Supplementary Fig S2, Table 1).

### Ink4a/Arf^*−*/*−*^ tenocytes produce 3D tendon-like constructs

**Figure 5:**
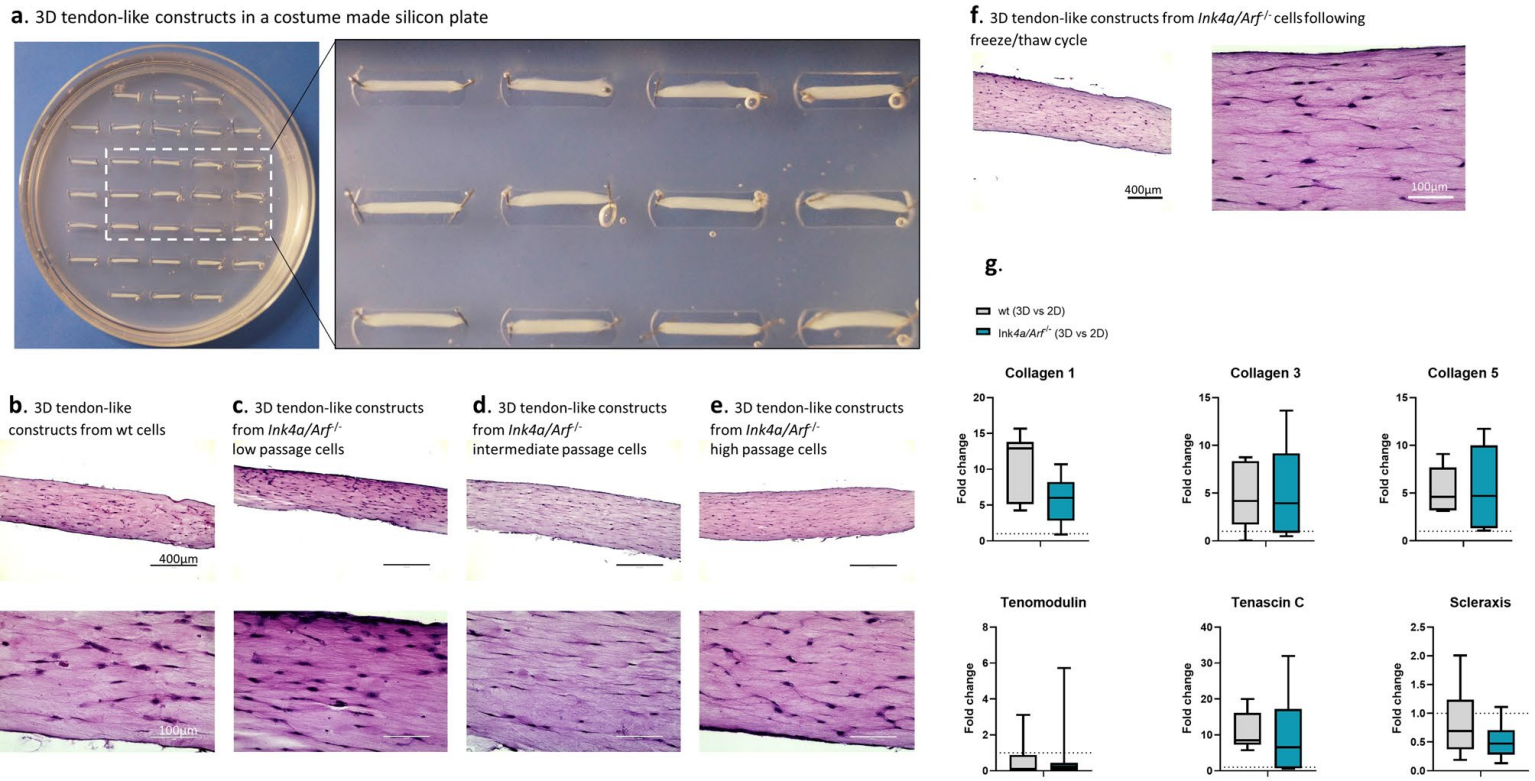
Tenocytes from *Ink4a/Arf*^*−*/*−*^ mice can produce 3D tendon-like constructs similar to wild type (wt) cells. (**a**) The macroscopic appearance of the silicone plate with the tendon-like constructs before harvesting (left) with a close-up of the tendons in their wells (right). (**b**)–(**e**) Histology of H&E stained tendon-like constructs obtained from wild-type (wt) cells in a low passage (**b**) and *Ink4a/Arf*^*−*/*−*^ cells in low (**c**), intermediate (**d**) and high (**e**) passages (top row: scale bar 400 µm, bottom row: scale bar 100 µm). (**f**) 3D tendon-like constructs of *Ink4a/Arf*^*−*/*−*^ tenocytes in an intermediate passage following freeze and thaw cycle showed analogous cell and fiber arrangement to 3D tendon-like constructs of wt or *Ink4a/Arf*^*−*/*−*^ cells, which had not undergone a freeze–thaw cycle (left micrograph: scale bar = 400 µm, right micrograph: scale bar = 100 µm). (**g**) qPCR analysis of tendon markers- *Col1*, *Col3, Col5, Scx, Tnmd* and *Tnc* presented as fold change (FC) in gene expression between 3 and 2D, for wt and *Ink4a/Arf*^*−*/*−*^ cells individually. The dotted line in each graph represents a FC of 1 (no difference between 2 and 3D gene expression). The expression in *Col1*,* Col3*,* Col5* and *Tnc*, but not in *Tnmd* and *Scx*, was significantly higher in 3D than 2D culture for both wt and *Ink4a/Arf*^*−*/*−*^ cells (for mean normalized mRNA expression and p-values, see Table 2).

To test the functional properties, *Ink4a/Arf*^*−*/*−*^ tenocytes of all passages were evaluated in 3D tendon-like constructs. Wild-type cells served as control and verified the comparable architecture (Fig. 5a).

Histological analysis confirmed the tendon structure of the 3D constructs characterized by a longitudinal arrangement of extracellular matrix (ECM) with cells scattered therein arranged parallel to the fibers irrespective of the phenotype and passage number (Fig. 5b–e). This remained unchanged after freeze/thaw cycles (Fig. 5f). As expected, and in line with the literature, tendon markers *Col1*, *Col3*, *Col5*, and *Tnc* were significantly higher expressed in the 3D tendon-like constructs than in 2D monolayer culture (Fig. 5g, Table 2). The expression of *Tnmd* and *Scx* was comparable between 2D- and 3D-cultured cells. No difference was noted between the wt and the cell line in expression of the tendon markers in the 3D tendon-like constructs.

**Table 2:** qPCR analysis of tendon markers expression in wild-type (wt) and *Ink4a/Arf*^*−*/*−*^ tenocytes in 2D and 3D culture.

2D wt (low passage) (mean ± s.d)	3D wt (low passage) (mean ± s.d)	Significance wt 3D\2D	2D *Ink4a/Arf*^*−*/*−*^ (all passages) (mean ± s.d)	3D *Ink4a/Arf*^*−*/*−*^ (all passages) (mean ± s.d)	Significance *Ink4a/Arf*^*−*/*−*^ 3D\2D
8.2 ± 4.7	87.6 ± 38.3	****	5.4 ± 5.1	30.4 ± 17.2	**
2.2 ± 1.4	10.5 ± 7.6	*	1.4 ± 1.7	7.6 ± 7.0	*
0.2 ± 0.1	1.01 ± 0.46	***	0.1 ± 0.1	0.6 ± 0.5	**
0.1 ± 0.001	0.05 ± 0.1	NS	0.03 ± 0.03	0.04 ± 0.08	NS
0.1 ± 0.1	1.4 ± 0.7	***	0.06 ± 0.06	0.63 ± 0.68	*
0.01 ± 0.01	0.01 ± 0.01	NS	0.01 ± 0.01	0.01 ± 0.003	NS

Values are shown as mean ± s.d. normalized mRNA levels. 3D tendon-like constructs and 2D monolayer culture were compared for each cell type using a t-test, and the *p*-value is shown for each comparison. For the *Ink4a/Arf*^*−*/*−*^ tenocytes, cells from low, intermediate, and high passage were combined.

Significance between 3 and 2D as follows- *p-value < 0.05, **p-value < 0.01, ***p-value < 0.001, ****p-value < 0.0001. *NS* not significant.

## Discussion

The current study successfully established an immortalized *Ink4a/Arf*^*−*/*−*^ tenocyte cell line, which combines a long lifespan with a stable tenogenic phenotype and high proliferation capacity. The tenocytes isolated from the Achilles tendons of *Ink4a/Arf*^*−*/*−*^ mice maintain their proliferation potential and tenogenic fusiform morphology even in long-term culture (passage 44 compared to a maximum of 5 passages for wt tenocytes) and express significantly lower levels of the senescence marker ß-gal compared to wt tenocytes. *Ink4a/Arf*^*−*/*−*^ cells express tendon markers and create 3D tendon-like constructs analogous to their wt counterparts. Based on these results, we conclude that deletion of the *Ink4a/Arf* locus does not interfere with tenogenic features. Furthermore, *Ink4a/Arf*^*−*/*−*^ cells also preserve their viability and morphology following freezing and thawing, facilitating their use for research purposes.

Immortalization, or the ability to avoid senescence, is crucial in overcoming the finite in vitro proliferative potential of primary mammalian cells, the so-called Hayflick limit. Immortalization is frequently accompanied by the deregulation of tumour suppressors and thus bears the potential risk of facilitating malignant transformation^29^. The *Ink4a/Arf* locus is one of the most frequently mutated sites in human cancer^16,18^. In the current study, no tumorigenesis was observed. As the mice included in this study were young, further studies evaluating the effect of p16Ink4a/Arf deletion on tumorigenesis in old mice are warranted.

*Ink4a/Arf*^*−*/*−*^ tenocytes do not undergo senescence and continue to proliferate for many passages (passage 44) with a steady expression of tendon markers. In contrast to previous studies reporting an early increase in proliferation rates in *Ink4a/Arf*^*−*/*−*^ embryonic fibroblasts^19,20^, *Ink4a/Arf*^*−*/*−*^ tenocytes proliferated at a comparable rate in low passages as wt tenocytes. Growth rates increased significantly in intermediate and high passages while the cells preserved their tenogenic characteristics.

In addition to the improved lifespan of the tenocytes cell line, the *Ink4a/Arf*^*−*/*−*^ cells maintained their tenogenic morphology, tendon marker expression and the ability to form 3D tendon-like constructs comparable to primary wt cells. Identifying and validating a tenocyte phenotype is hindered by the lack of tendon-specific markers as the tendon ECM genes such as *Col1, Col3, Col5* and *Tnc* are also expressed in other musculoskeletal tissues^14,30^. Therefore, a panel of markers, including not only ECM genes but also genes associated with tendon development, such as *Scx* and *Tnmd*, are required to specify tenogenic characteristics^14,30^. In the current study, common tendon markers (*Col1, Col3, Col5, Scx, Tnmd* and *Tnc*) were expressed in *Ink4a/Arf*^*−*/*−*^ cells of all passages equivalent to wt cells. This finding is important since previous tenocyte cell lines showed a significant reduction in tendon markers gene expression compared to wt tenocytes^11^. Flow cytometry is less frequently used for tendon characterization since the markers are more established for progenitor cells (e.g. mesenchymal stem cells or tendon progenitor cells) or fibroblasts in general^28,31–34^. However, although mesenchymal stem cells and tenocytes are different cell types, similar fibroblastic surface marker expression has previously been reported^34,35^ and adds another valuable tool to define a tenogenic phenotype. In our study, primary wt cells and *Ink4a/Arf*^*−*/*−*^ tenocytes in all passages highly (> 95%) expressed the markers CD29 and CD44, but CD90 and CD105 expression decreased with passaging. This finding supports a previous report of murine mesenchymal stem cells expressing CD105 to a similar percentage with decreasing CD105 expression during passaging^36^. Regarding the loss of CD90 expression during passaging, the literature is equivocal with some studies reporting a decrease of CD90 expression in stem cells with passaging, while others did not observe the same phenomenon^24,34,37,38^.

In recent years, 3D models have gained popularity since they provide a higher resemblance of the morphological and functional properties of the tissue as compared to monolayer cell culture^10,39–41^. In the current study, the creation of 3D tendon-like constructs was essential to assess the function of the cells as tenocytes align to the direction of the anchor points, contract and produce ECM. The *Ink4a/Arf*^*−*/*−*^ cells formed those constructs in all passages within the same time frame (10 days) as wt tenocytes, demonstrating their tenogenic function. *Col1, Col3, Col5 and Tnc* gene expressions revealed the tendon markers expression is significantly higher in 3D compared to 2D monolayer. This tendency was not shown in *Scx* and *Tnmd,* which revealed a similar, very low expression in both 2D and 3D. The higher expression of tendon markers in the 3D tendon-like constructs emphasizes the importance of studying tenocytes in systems resembling the natural environment, which supports the full functionality of the cells.

In summary, we successfully established an immortalized tenocyte cell line isolated from the Achilles tendon of *Ink4a/Arf* depleted mice, which maintains tenocytes’ morphological and functional features. Compared to wt cells, the *Ink4a/Arf*^*−*/*−*^ cells have higher proliferation rates and lower senescence, while the expression of tendon markers, morphology, and ability to create 3D tendon-like constructs remain similar to wt tenocytes. In addition, *Ink4a/Arf*^*−*/*−*^ tenocytes can reach high passage numbers and withstand freeze–thaw cycles while maintaining tenogenic characteristics. Combining a long lifespan in vitro with a stable tenogenic phenotype, the *Ink4a/Arf*^*−*/*−*^ tenocyte cell line will enable more extended experiments and significantly facilitate research into the highly prevalent, disabling tendon diseases. Notably, the phenotypically stable *Ink4a/Arf*^*−*/*−*^ tendon cell line will also limit the number of animals needed for each experiment and enable the development of in vitro alternatives to animal trials.

## Methods

### Mice

C57BL/6N (wild-type = wt, n = 3) and *Ink4a/Arf*-deficient (*Ink4a/Arf*^*−*/*−*^, n = 3)^20^ female mice were housed under Specific-pathogen-free (SPF) conditions at the University of Veterinary Medicine Vienna, Austria. All experiments were conducted in 8- to 12-week-old mice. Mice were placed under general anesthesia with isoflurane inhalation and euthanized using cervical dislocation for reasons unrelated to this study. Both Achilles tendons were harvested immediately following euthanasia.

Experiments were approved by the institutional ethics and animal welfare committee and the national authority (license BMWF-68.205/0218-II/3b/2012), in accordance with ARRIVE guidelines. All methods were performed in accordance with the relevant guidelines and regulations implemented at the University of Veterinary Medicine Vienna, the Institutional Ethics Committee (“Ethics and Animal Welfare Committee”) of the University of Veterinary Medicine Vienna.

### Tenocytes isolation and cell culture

The Achilles tendons of both hind limbs were harvested, washed with phosphate-buffered saline (PBS), and sectioned into 5 mm pieces following removal of the paratenon. Tenocytes were isolated by enzymatic digestion using 3 mg/mL Collagenase Type II (Gibco Lifetechnologies, Vienna, Austria) in culture media (minimal essential medium) supplemented with 10% fetal bovine serum (FBS), 1% L-Glutamine, 100 U/mL penicillin and 0.1 mg/mL Streptomycin for 6 h. After washing with PBS (centrifugation for 5 min at 400 g), cells were cultivated in culture media at 37 °C and 5% CO_2_ until the desired passage and number of cells were obtained.

Experiments were performed for the wt cells in a low passage (passage 2–5) and the *Ink4a/Arf*^*−*/*−*^ cells in 3 different passages—low (passage 2–5), intermediate (passage 14–17) and high (passage 35–44).

All experiments were carried out with biological triplicates (the same three wt and three *Ink4a/Arf*^*−*/*−*^ mice for all experiments).

For freeze–thaw cycle assessment, *Ink4a/Arf*^*−*/*−*^ cells were frozen in 10% DMSO and 90% FBS at an intermediate passage and were used for experiments 2–3 passages following thawing.

### Western blot analysis

Western blotting (WB) was performed using standard protocols^42^. Cells from *Ink4a/Arf*^*−*/*−*^ mice at intermediate passage were compared to wt cells. Total protein extraction was done with Laemmli lysis buffer, and protein concentration was determined using a BCA assay (Thermo scientific No. 23225). Proteins were separated on an SDS–polyacrylamide electrophoresis (PAGE) gel and transferred to PVDF membranes in an overnight wet transfer. Membranes were incubated overnight at 4 °C with primary antibodies, followed by 1 h room temperature incubation with secondary antibodies using monoclonal rabbit anti-mouse p16INKa (Abcam, ab211542; dilution 1: 2,000), monoclonal chicken anti-mouse α-Tubulin (DM1A, Sigma-Aldrich, CP06; dilution 1: 1000). Chemiluminescence detection was performed using the Bio-Rad ChemiDoc Imaging systems.

### Proliferation assay

Cells were plated in 96-well plates (3000 cells/well). The proliferation rate was measured via DNA fluorescence using the CyQuant assay (Invitrogen, C35012) 24, 48, 72 and 96 h after seeding.

### Cellular senescence/β-galactosidase activity

Cells were plated in 96-well plates (3000 cells/well). Cellular senescence was quantified by measuring senescence-associated β-galactosidase (SA-β-gal) activity according to the manufacturer’s instructions (SA-β-gal activity, Cell Biolabs, CBA-231) 48 h after seeding.

### Morphology

Cells were imaged at 60–70% confluency using the EVOS FL Auto imaging system in phase contrast with a 40 × objective (ThermoFisher Scientific, AMEP4680).

Cell area and circularity were measured as previously described^43^. Shortly, the boundaries of individual cells were manually traced on the images using ImageJ 1.53t software to calculate the cell area and the perimeter of each cell. Circularity was defined as C = 4π(A/P2), where P is the perimeter, and A is the cell area. Circularity value of 1 indicates a perfect circle while value of 0 indicates an elliptical shape.

### Flow cytometry

Cells (1 × 10^5^) were stained with fluorophore-conjugated antibodies for surface markers CD29, CD44, CD90 and CD105^28^ (CD29-PE (HMb1-1; eBioscience), CD44-PE (IM7; eBioscience), CD90-FITC (30-H12; BioLegend), CD105-PE (MJ7/18; eBioscience)) diluted 1:200 in PBS with 2% FBS and incubated for 30 min at 4 °C. Cells were washed twice with PBS and measured on a BD FACS Canto II. To detect apoptotic cells, tenocytes were resuspended in 1 × Annexin-V binding buffer and stained with Annexin-V for 15 min at room temperature according to the manufacturer’s instructions (eBioscience, 88-8005-74). To analyze the DNA content, the respective cells were stained with DAPI (D9542, Sigma). Therefore, 1 × 10^5^ cells were washed with PBS, resuspended in 2% paraformaldehyde in PBS and incubated for 10 min at 37 °C. After centrifugation, the supernatant was discarded and cells were permeabilized by 90% methanol in PBS with 2% FCS and 0.2% Tween-20 on ice for 30 min. Cells were washed twice and stained with 2 µg/ml DAPI in PBS with 2% FCS and 0.2% Tween-20 for 10 min on ice followed by washing twice. Data was acquired on a Beckman Coulter Cytoflex S. Flow cytometry data was analyzed by BD FACSDiva Software or FlowJo (v10.6.1).

### RNA extraction and quantitative real time-polymerase chain reaction (qRT-PCR)

Tenocytes were seeded in 12-well plates (1 × 10^5^ cells/well). After 24 h, RNA was isolated using Trizol and Chloroform in a ratio of 5–1. Total RNA was recovered by adding isopropanol and glycerol. The mixture was placed on ice and centrifuged for 60 min at 13,000 rpm. The total RNA pellet was washed twice with 75% ethanol and solubilized in RNase-free water. DNA remains were removed by a DNA removal kit (Life Technologies). 5 ng RNA was used for the qPCR reaction (qPCR One-Step Eva Green kit, Bio&amp; Sell, Feucht, Germany). The results were analyzed using the QuantStudio (Invitrogen, Thermo Fischer Scientific) software. The mRNA levels were calculated by first normalizing using the BestKeeper Index (Housekeeping genes used- *GAPDH*, *36B4*, *Rps18*) and then using the 2^^ct∆^ method. All primers (Supplementary Table S3) were designed using the Primer3 software.

### 3D tendon-like constructs

Tendon-like constructs (1 cm long) were produced by providing tenocytes suspended in *Collagen type 1* gel with anchor points embedded into the wells (well dimension- 10 mm long, 3 mm wide and 2 mm deep) of a 31-well silicon plate. The 31-well plate was custom-made using silicon (Sylgard 184 Silicone elastomer Dow Corning) and a 3D-printed template (Supplementary Fig S4). After autoclaving, the silicone plate was placed in a 100/20 mm cell culture dish, and each well was coated with a biocompatible polymer (Lipidure, AMS biotechnology) to prevent attachment of the cells to the silicone. Tenocytes were suspended in a 60% α-MEM/ 40% collagen (PureCol EZ Gel solution, Sigma-Aldrich) mixture kept on ice. Sixty μl of the solution containing 2.5 × 10^4^ cells were added to each well. Following 2 h of incubation at 37 °C and 5% CO_2_ to allow the gel to solidify, 2 pins (Austerlitz insect’s pins, 0.2 mm) were inserted into each tendon at the edge of each well and culture medium was added. Culture medium was replaced every 3 days. 3D tendon-like constructs formed gradually over a few days, during which the cells and collagen arranged longitudinally. By day 10, the 3D tendon-like constructs were visible and stable. The 3D tendon-like constructs were analyzed by qRT-PCR and histology.

For qRT-PCR, 31 of the 3D tendon-like constructs were pooled and RNA was extracted as described earlier. Analysis was done as a fold change (FC) of the 3D tendon-like construct expression relative to the average 2D (monolayer) expression. A fold change higher than 1 will mean higher expression of the marker in the 3D tendon-like construct in comparison to the 2D monolayer. The low, intermediate, and high passages of the *Ink4a/Arf*^*−*/*−*^ groups were pooled together into one group named *Ink4a/Arf*^*−*/*−*^.

### Histology

For histology, the 3D tendon-like constructs were washed with PBS and fixed with formalin solution while still in the silicone plate. Following formalin fixation, the 3D tendon-like constructs were removed, dried, and embedded in paraffin. Paraffin-embedded 2-µm sections were stained with hematoxylin and eosin G. Micrographs of the 3D tendon-like constructs were taken using the EVOS FL Auto imaging system in colour phase with a 100 × and 400 × objective (ThermoFisher Scientific, AMEP4680).

### Statistical analysis

Statistical analyses were performed using Graph Pad Prism software (version 8.4.3).

Continuous variables were expressed as mean ± standard deviation (s.d.), and categorical variables were expressed as percentages. Differences between wt and *Ink4a/Arf*^*−*/*−*^ cells and between the different passages of *Ink4a/Arf*^*−*/*−*^ cells were analyzed using a t-test or ANOVA with Tukey's multiple comparisons test. A *p*-value < 0.05 was considered significant.

## Supplementary Information

Supplementary Information.

## Data Availability

All data generated or analyzed during this study are included in this published article (and its Supplementary Information files).

## References

[1] Thomopoulos S, Parks WC, Rifkin DB, Derwin KA (2015). Mechanisms of tendon injury and repair. J. Orthop. Res..

[2] Walden G (2017). A clinical, biological, and biomaterials perspective into tendon injuries and regeneration. Tissue Eng. Part B Rev..

[3] Abraham T, Koob K, Carkaci-Salli N, Aydogan U (2019). Detection of the tendon properties in posterior tibial tendinopathy in three-dimensional space using high resolution multiphoton and second harmonic generation imaging. Foot Ankle Orthop..

[4] Nichols AEC, Best KT, Loiselle AE (2019). The cellular basis of fibrotic tendon healing: Challenges and opportunities. Transl. Res..

[5] Millar NL (2021). Tendinopathy. Nat. Rev. Dis. Primers.

[6] Djalali-Cuevas A (2018). Rna sequencing and meta-profiling for a better understanding of the musculoskeletal system biology. Orthop. Proc..

[7] Longo UG, Ronga M, Maffulli N (2018). Achilles tendinopathy. Sports Med. Arthrosc. Rev..

[8] Bottagisio M, Lovati AB (2017). A review on animal models and treatments for the reconstruction of Achilles and flexor tendons. J. Mater. Sci. Mater. Med..

[9] Kendal, A. R. *et al. Identification of human tendon cell populations in healthy and diseased tissue using combined single cell transcriptomics and proteomics*. 2019.12.09.869933. 10.1101/2019.12.09.869933 (2019).

[10] Shimada A (2014). Efficient expansion of mouse primary tenocytes using a novel collagen gel culture method. Histochem. Cell Biol..

[11] Denduluri SK (2016). Immortalized mouse achilles tenocytes demonstrate long-term proliferative capacity while retaining tenogenic properties. Tissue Eng. Part C Methods.

[12] Yao L, Bestwick CS, Bestwick LA, Maffulli N, Aspden RM (2006). Phenotypic drift in human tenocyte culture. Tissue Eng..

[13] Schulze-Tanzil G (2004). Cultivation of human tenocytes in high-density culture. Histochem. Cell Biol..

[14] Jo CH, Lim H-J, Yoon KS (2019). Characterization of tendon-specific markers in various human tissues, tenocytes and mesenchymal stem cells. Tissue Eng. Regen. Med..

[15] Boraschi D, Li D, Li Y, Italiani P (2021). In vitro and in vivo models to assess the immune-related effects of nanomaterials. Int. J. Environ. Res. Public Health.

[16] Sharpless NE, DePinho RA (1999). The INK4A/ARF locus and its two gene products. Curr. Opin. Genet. Dev..

[17] Baker DJ (2008). Opposing roles for p16 Ink4a and p19 Arf in senescence and ageing caused by BubR1 insufficiency. Nat. Cell Biol..

[18] Sharpless NE, Ramsey MR, Balasubramanian P, Castrillon DH, DePinho RA (2004). The differential impact of p16 INK4a or p19 ARF deficiency on cell growth and tumorigenesis. Oncogene.

[19] Sharpless NE (2001). Loss of p16 Ink4a with retention of p19 Arf predisposes mice to tumorigenesis. Nature.

[20] Serrano M (1996). Role of the INK4a locus in tumor suppression and cell mortality. Cell.

[21] Carnero A, Hudson JD, Price CM, Beach DH (2000). p16 INK4A and p19 ARF act in overlapping pathways in cellular immortalization. Nat. Cell Biol..

[22] Kim WY, Sharpless NE (2006). The regulation of INK4/ARF in cancer and aging. Cell.

[23] Zindy F, Quelle DE, Roussel MF, Sherr CJ (1997). Expression of the p16INK4a tumor suppressor versus other INK4 family members during mouse development and aging. Oncogene.

[24] Kohler J (2013). Uncovering the cellular and molecular changes in tendon stem/progenitor cells attributed to tendon aging and degeneration. Aging Cell.

[25] Xu H, Liu F (2018). Downregulation of FOXP1 correlates with tendon stem/progenitor cells aging. Biochem. Biophys. Res. Commun..

[26] Han W, Wang B, Liu J, Chen L (2017). The p16/miR-217/EGR1 pathway modulates age-related tenogenic differentiation in tendon stem/progenitor cells. Acta Biochim. Biophys. Sin..

[27] Tsutsui T (2002). Association of p16 INK4a and pRb inactivation with immortalization of human cells. Carcinogenesis.

[28] Stolk M (2017). New insights into tenocyte-immune cell interplay in an in vitro model of inflammation. Sci. Rep..

[29] Wang Y, Chen S, Yan Z, Pei M (2019). A prospect of cell immortalization combined with matrix microenvironmental optimization strategy for tissue engineering and regeneration. Cell Biosci..

[30] Jelinsky SA, Archambault J, Li L, Seeherman H (2010). Tendon-selective genes identified from rat and human musculoskeletal tissues. J. Orthop. Res..

[31] Wu YF, Chen C, Tang JB, Mao WF (2020). Growth and stem cell characteristics of tendon-derived cells with different initial seeding densities: An in vitro study in mouse flexor tendon cells. Stem Cells Dev..

[32] Lee K, Clegg P, Comerford E, Canty-Laird E (2015). The stem cell niche in tendon and ligament: Investigating alterations with ageing and disease. Orthop. Proc..

[33] Chen W (2015). Dexamethasone inhibits the differentiation of rat tendon stem cells into tenocytes by targeting the scleraxis gene. J. Steroid Biochem. Mol. Biol..

[34] Lui PPY (2015). Markers for the identification of tendon-derived stem cells in vitro and tendon stem cells in situ—update and future development. Stem. Cell Res. Ther..

[35] Lee KJ, Clegg PD, Comerford EJ, Canty-Laird EG (2018). A comparison of the stem cell characteristics of murine tenocytes and tendon-derived stem cells. BMC Musculoskelet. Disord..

[36] Anderson P, Carrillo-Gálvez AB, García-Pérez A, Cobo M, Martín F (2013). CD105 (Endoglin)-negative murine mesenchymal stromal cells define a new multipotent subpopulation with distinct differentiation and immunomodulatory capacities. PLoS ONE.

[37] Tan Q, Lui PPY, Rui YF (2012). Effect of in vitro passaging on the stem cell-related properties of tendon-derived stem cells—implications in tissue engineering. Stem Cells Dev..

[38] Zhou Z (2010). Tendon-derived stem/progenitor cell aging: Defective self-renewal and altered fate. Aging Cell.

[39] Gehwolf R (2019). Global responses of Il-1β-primed 3D tendon constructs to treatment with pulsed electromagnetic fields. Cells.

[40] Duval K (2017). Modeling physiological events in 2D vs. 3D cell culture. Physiology.

[41] Barsby T, Bavin EP, Guest DJ (2014). Three-dimensional culture and transforming growth factor beta3 synergistically promote tenogenic differentiation of equine embryo-derived stem cells. Tissue Eng. Part A.

[42] Maurer B, Brandstoetter T, Kollmann S, Sexl V, Prchal-Murphy M (2020). Inducible deletion of CDK4 and CDK6—deciphering CDK4/6 inhibitor effects in the hematopoietic system. Haematologica.

[43] Inguito KL (2022). Stress deprivation of tendon explants or Tpm3.1 inhibition in tendon cells reduces F-actin to promote a tendinosis-like phenotype. MBoC.

