# Supplementary material for: Immortalized murine tenocyte cells: a novel and innovative tool for tendon research
Source: Sci Rep. 2023 Jan 28;13:1566. doi: 10.1038/s41598-023-28318-4 (PMC9884217; doi:10.1038/s41598-023-28318-4)
Supplement: Supplementary file 1 — Supplementary Information. [file 41598_2023_28318_MOESM1_ESM.pdf]

# Supplementary Information

# Immortalized murine tenocyte cells – a novel and innovative tool for tendon research

Gil Lola Oreff*[1,2], Barbara Maurer[2], Ahmed N. ELKhamary[1], Iris Gerner[1], Veronika Sexl[2**], Florien Jenner[1**]

[1] Veterinary Tissue Engineering and Regenerative Medicine Lab, Equine Surgery Unit, Department of Companion Animals and Horses, University of Veterinary Medicine Vienna, Veterinaerplatz 1, 1210 Vienna, Austria

[2] Institute of Pharmacology and Toxicology, Department of Biomedical Sciences, University of Veterinary Medicine Vienna, Veterinaerplatz 1, 1210 Vienna, Austria

* Corresponding author: gil.oreff@vetmeduni.ac.at

** Shared last author

**Supplementary Figure 1**

Fig S1:

a: Uncropped blot of immunoblot shown in Figure 1a.

b: Gating Strategy for DNA content/aneuploidy analysis via DAPI staining. A representative dot plot of an *Ink4a/Arf*$^{-/-}$ tenocyte cell line is shown.

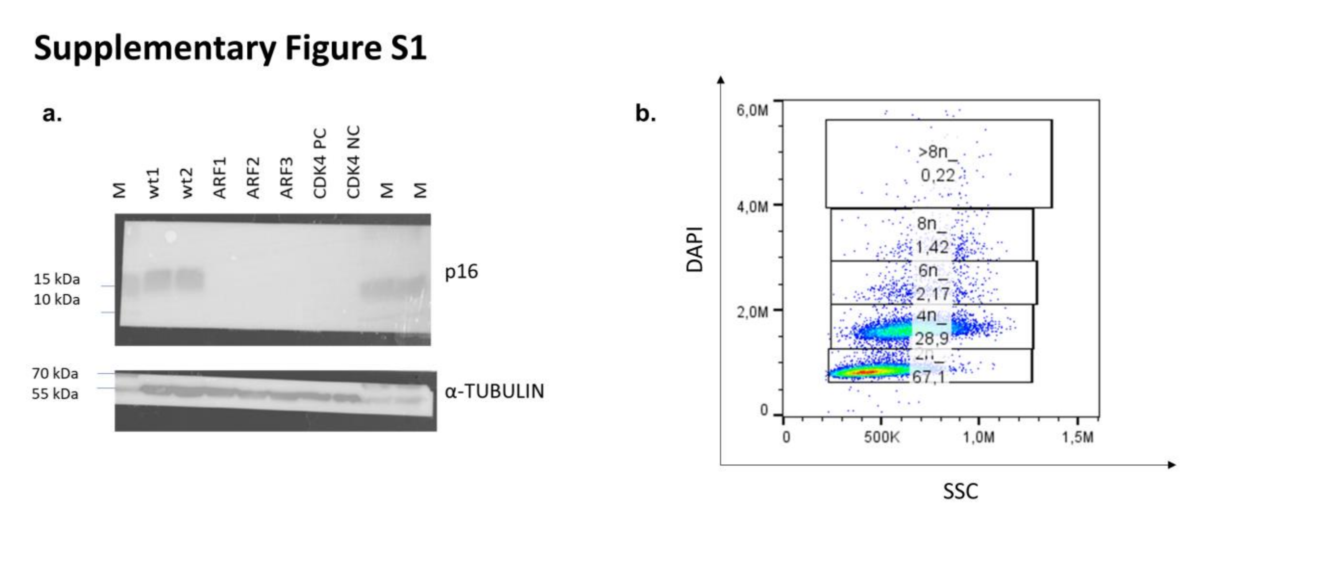

**Supplementary Figure 2**:

Fig S2: Flow cytometry analysis of CD29, CD105, CD90 and CD44 expression in wild-type (wt) and *Ink4a/Arf*$^{-/-}$ cells from different passages. The figure represents the number of live cells (%) expressing each marker in each cell type. n = 3 different biological replicates. ** p-value< 0.01

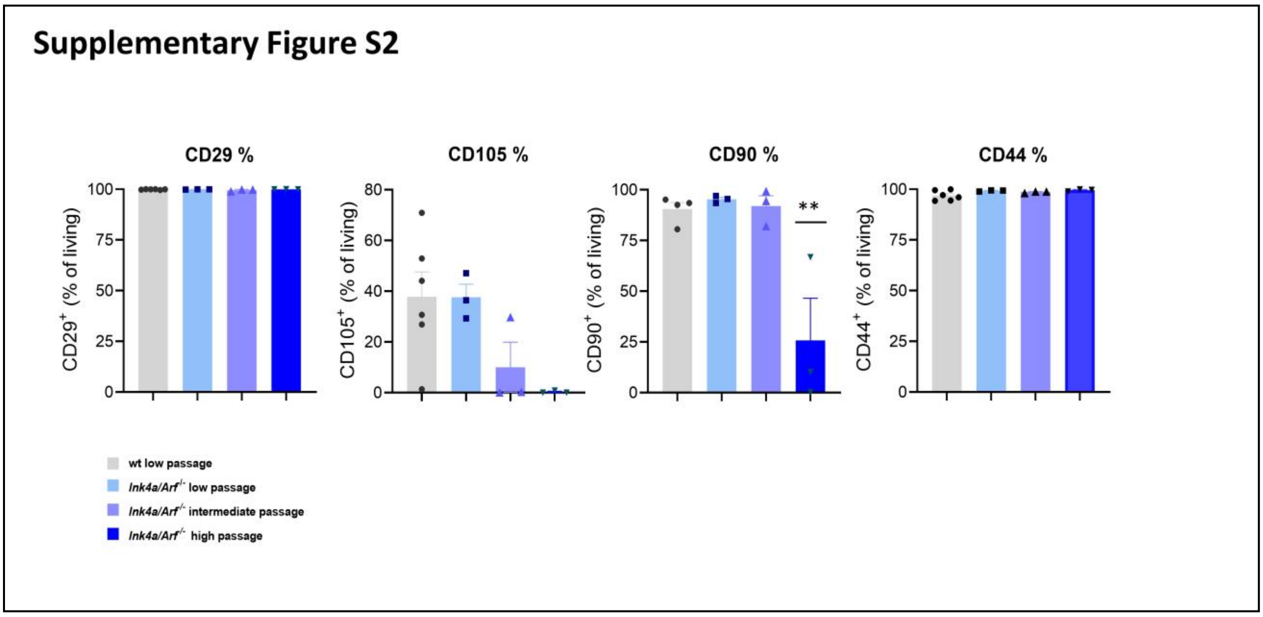

**Supplementary Figure 4**

Fig S4: Dimensions of the 3D printed silicone plate used for creation of 3D tendon-like constructs

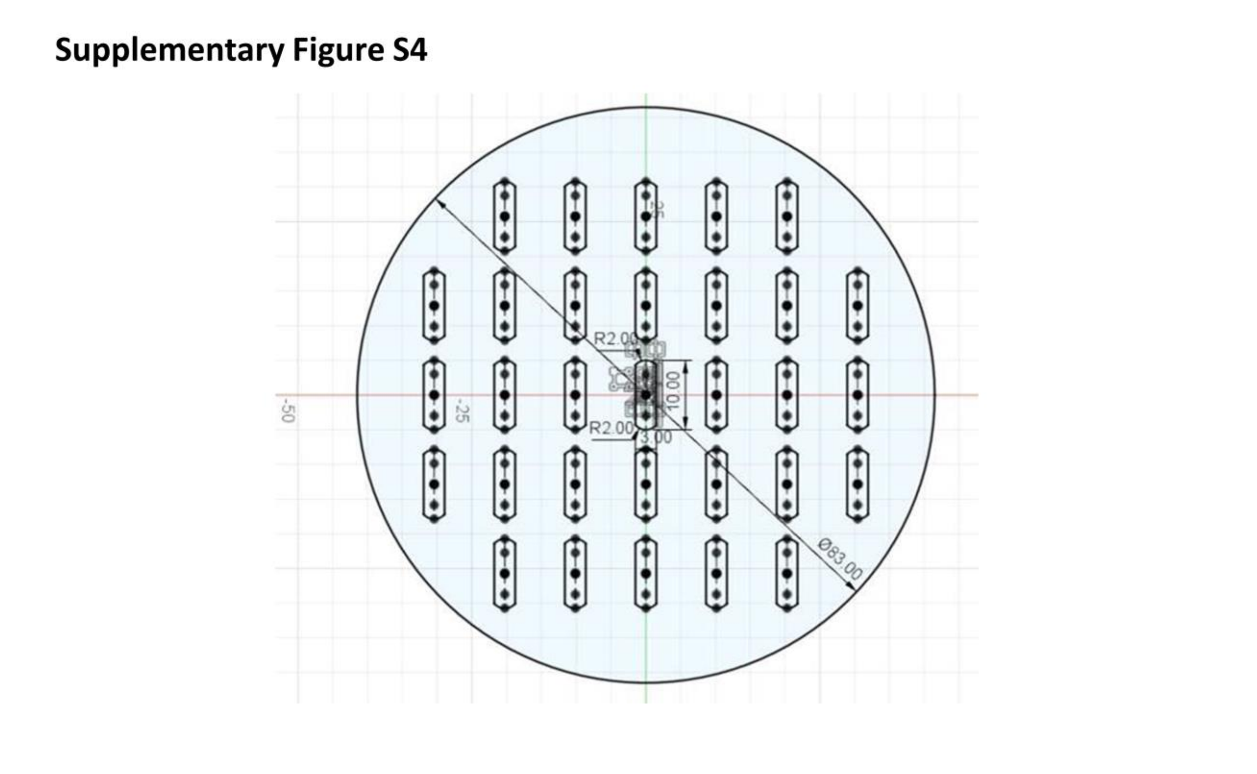


**Supplementary Table 3**

Table S3: RT-qPCR Primer sequences

| | Forward 5'-3' | Reverse 5'-3' |
|---|---|---|
| Collagen type 1 (*COL1*) | *GAGCGGAGAGTACTGGATCG* | *GCTTCTTTTCCTTGGGGTTC* |
| Collagen type 3 (*COL3*) | *CTGGAGATAAGGGTGAAGGT* | *GAGGGCCTCCTTCACCTTTCT* |
| Collagen type 5 (*COL5*) | *CAGTGAATTCAAGCGTGGGA* | *GTAGGTGACGTTCTGGTGGG* |
| Scleraxis (*SCX*) | *TTGAGCAAAGACCGTGACAGA* | *TGTGGACCCTCCTCCTTCTAAC* |
| Tenomodulin (*TNMD)* | *GTCACATTCTAAATGCAGAAG* | *CTCCCCCAAAACAGGACAAT* |
| Tenascin C (*TNC*) | *CTGCTGTCAAGGGAGACAAG* | *AGACACCCGTAAGTCCTTGG* |
| Glyceraldehyde-3-phosphate dehydrogenase (*GAPDH*) | *CTGCACCACCAACTGCTTAG* | *GTCTTCTGGGTGGCAGTGAT* |
| Rplp0 *(36b4)* | *AGATTCGGGATATGCTGTTGG* | *AAAGCCTGGAAGAAGGAGGTC* |
| Ribosomal protein S18 (*Rps18*) | *GCCGTTCTTAGTTGGTGGAG* | *GAACGCCACTTGTCCCTCTA* |
